# Ultrasound measurement of carotid flow time changes with volume status

**DOI:** 10.1186/cc13321

**Published:** 2014-03-17

**Authors:** DC Mackenzie, NA Khan, D Blehar, CP Stowell, VE Noble, AS Liteplo

**Affiliations:** 1 Maine Medical Center, Portland, ME, USA; 2 Massachusetts General Hospital, Boston, MA, USA; 3 University of Massachusetts, Worcester, MA, USA

## Introduction

Assessment of volume status and responsiveness guides resuscitation strategy. Non-invasive techniques are desirable. Changes in carotid flow time have been proposed as a marker of volume status, but few data support their use. We sought to determine whether carotid flow time decreased in the volume-depleted state of acute blood loss, and whether volume-depleted individuals would demonstrate an increase in carotid flow time after a passive leg raise (PLR) maneuver.

## Methods

Volunteers aged 18 to 55 presenting to the hospital's blood donor center for whole blood donation were eligible to participate. Individuals with a history of aortic or carotid artery disease, atrial fibrillation, or a contraindication to blood donation were excluded. Prior to blood donation, an investigator performed an ultrasound of the right common carotid artery with a high-frequency linear transducer, obtaining a Doppler tracing of carotid artery flow. Measurements of peak velocity, systole time, and carotid flow time were obtained. A PLR was performed for 30 seconds, followed by repeat measurements of carotid velocity and flow time. Whole blood was then collected according to the blood donor center's protocol. Immediately after blood donation, repeat measurements of carotid flow and velocity were obtained in the supine position and after a PLR. Carotid flow times corrected for heart rate (FTc) were analyzed with Student's *t *test. The institutional review board approved the study.

## Results

Eighty donors were screened for participation by two investigators; 68 consented and completed donation (60.3% female, mean age 31). Donors had mean blood loss of 450 ml. The mean FTc supine after blood donation was 296 ms; this was significantly different from the FTc prior to donation (supine = 320 ms, PLR = 323 ms; *P *< 0.0001). The mean FTc following blood donation and PLR was 321 ms, significantly different from the supine position after donation (*P *< 0.0001), but not pre-donation measurements.

## Conclusion

Ultrasound measurement of carotid flow time was significantly decreased in the setting of acute blood loss. An autobolus by PLR after blood loss restored FTc to pre-donation levels. Further investigation of FTc as a non-invasive predictor of volume responsiveness is warranted.

